# Cellular Senescence Triggered by Food and Environmental Genotoxins

**DOI:** 10.3390/ijms27052389

**Published:** 2026-03-04

**Authors:** Bernd Kaina, Maja T. Tomicic, Markus Christmann

**Affiliations:** Institute of Toxicology, University Medical Center of the Johannes Gutenberg University Mainz, Obere Zahlbacher Str. 67, D-55131 Mainz, Germany; tomicic@uni-mainz.de

**Keywords:** senescence, genotoxic agents, food carcinogens, environmental carcinogens, senescence pathways, DNA damage and repair, N-nitrosamines, red meat, food contaminants, cancer

## Abstract

Cellular senescence (CSEN) is caused by a variety of factors that trigger complex molecular pathways. These include telomere shortening, oncogene activation and replicative stress, as well as DNA damage caused by genotoxic anticancer drugs and endogenous and exogenous genotoxins. Here, we review the induction of CSEN by exogenous genotoxic insults resulting from food and environmental exposures. The available data show that genotoxins/carcinogens in tobacco smoke and smokeless tobacco, in the environment, in food, beverages and life-style products induce CNS. The exposures include N-nitroso compounds, polycyclic aromatic hydrocarbons, heterocyclic aromatic amines, acrylamide, heavy metals, fine dust, mycotoxins, phytotoxins, and phycotoxins. Also, heme in red meat contributes to CSEN as it catalyzes the formation of genotoxic species in the colon. Induction of CSEN by external genotoxins/carcinogens is bound on the DNA damage response pathway (DDR), which relies on activation of the ATM/ATR-CHK2/CHK1-p53-p21 axis and the p53-independent p16/p14 axis, eliciting cyclin-dependent kinase inhibition and permanent cell cycle arrest. Other factors that can be involved are DREAM, MAPK, cGAS/Sting, and NF-κB. The accumulation of non-repaired DNA damage triggering CSEN following external genotoxic exposures may contribute significantly to the amelioration of senescent cells and organ failure with age in humans. Senescent cells drive, via the senescence-associated secretory phenotype (SASP), inflammation that is involved in many diseases, including cancer. Although most of the studies were performed with in vitro cell systems, the consequences of CSEN induction by genotoxic nutritional components and environmental exposures seem to be underestimated. Since CSEN correlates with aging, it is reasonable to conclude that exogenous genotoxic pollutants contribute significantly to the aging process through CSEN induction. In light of these findings, it is deduced that reducing genotoxin exposures and using “rejuvenation” supplements (senotherapeutics) are reasonable strategies to counteract cellular senescence and the aging process.

## 1. Introduction

Chemical genotoxins/carcinogens are present in the environment, in food and beverages and life-style-related products (tobacco, alcohol, cosmetics) [1,2]. Furthermore, they can be produced endogenously in the body by the cellular metabolism, bacterial activity and activated immune cells such as monocytes and macrophages, producing free radicals [3]. Additionally, physical carcinogens like sunlight [4] and ionizing radiation [5], as well as many pharmaceuticals, notably the classical anticancer drugs [6], are of importance. All these carcinogens attack the DNA, and as a result, diverse DNA lesions are induced, which are either neutral or cause mutations, chromosomal changes, malignant transformation, cellular aging, or cell death. For a long time, the focus of research was on the induction of mutations and cell death. Cell death is important as it counteracts the long-term adverse effects of genotoxic stress by eliminating cells with damaged DNA. It is also important in regulating tissue homeostasis following genotoxic stress in cells having a high turnover, like the immune system, where endogenous DNA damage triggers the death of activated immune cells [7]. Death by apoptosis and other mechanisms is a desired process when cancer cells are killed, but an undesirable process when anticancer drugs kill cells in the normal tissue.

In recent years, we have learned that DNA damage causes not only cell death, but also cellular senescence (CSEN). CSEN refers to a property of cells to permanently cease proliferation. This occurs when the cell division potential of normal cells is exhausted (replicative senescence) or when cells are stressed by increased proliferation signaling, leading to DNA replication stress (oncogenic senescence). CSEN can also be caused, similar to cell death, by various genotoxic compounds to which we are exposed in normal life (sunlight, ionizing radiation, and carcinogens in the environment and food) or during cancer therapy (radiation and genotoxic chemotherapeutics). The induction of CSEN means that cells are not killed but rather enter a dormant state. This state is characterized by sustained activation of intracellular signaling pathways that inhibit cell cycle progression, block apoptosis pathways, and release inflammatory mediators (SASP). It is further characterized by global genomic reprogramming and metabolic changes [8].

In this review, we describe the mechanisms involved in genotoxin-induced CSEN and consider CSEN from the perspective of the potential of environmental genotoxins and food-borne carcinogens and food contaminants as inducers of CSEN.

## 2. DNA Damage Giving Rise to CSEN

**Figure 1:**
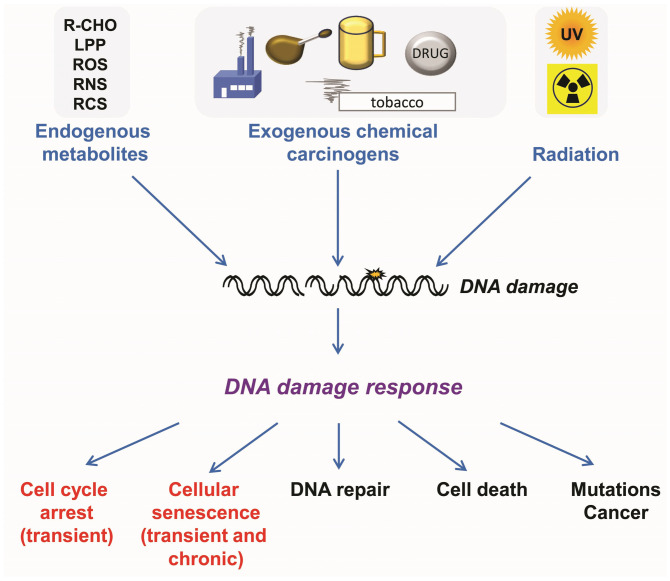
**Cellular responses triggered by endogenous and exogenous genotoxins.** Endogenous metabolites are aldehydes, lipid peroxidation products (LPP) (malondealdehyde, 4-hydroxynonenal, acrolein), reactive nitrogen species (RNS) (peroxynitrite, reactive oxygen species (ROS), reactive carbonyl species (RCS) (methylglyoxal, glyoxal). Exogenous genotoxins result from industrial pollutants, food, notably processed meat, and beverages, notably alcohol, tobacco, and pharmaceuticals. Physical exposures result from ultraviolet (UVA and UVB light) and ionizing radiation.

There are a plethora of insults that damage DNA and, consequently, various adverse effects are induced by them. Key is the activation of the DNA damage response (DDR) (Figure 1).

It is important to note that not all DNA lesions induced by genotoxic agents are harmful. Some are genetically neutral, like N7-methylguanine and alkyl phosphotriesters; some are still instructive but have mispairing properties, causing exchange mutations such as 8-oxo-guanine and O^6^-methylguanine (O^6^-MeG); some are tolerated by translesion synthesis, but the majority are non-instructive, being just a steric hindrance for the replication and transcription machinery. As a rule, it seems that all DNA adducts blocking replication are potentially cytotoxic and have the ability to trigger CSEN. To these belong adducts induced by simple alkylating agents, ultraviolet (UV) light, ionizing radiation (IR), polycyclic hydrocarbons, and others. A severe replication and transcription block results from interstrand crosslinks (ICLs) induced by some chemical weapons (mustards), anticancer drugs (cisplatin, cyclophosphamide, and melphalan), and other therapeutics such as psoralen-UV light [9]. Of note, there is an arsenal of natural compounds produced by plants that are genotoxic, and the responsible adducts are only partly known [10,11].

An intriguing question is whether DNA adducts themselves are able to activate cell death and CSEN pathways or whether DNA double-strand breaks (DSBs) resulting from them as repair intermediates or during blocked transcription and replication are the critical downstream players. It is generally accepted that DSBs and permanently blocked replication forks are strong activators of the DNA damage response (DDR), which is key in the regulation of CSEN pathways.

**Figure 2:**
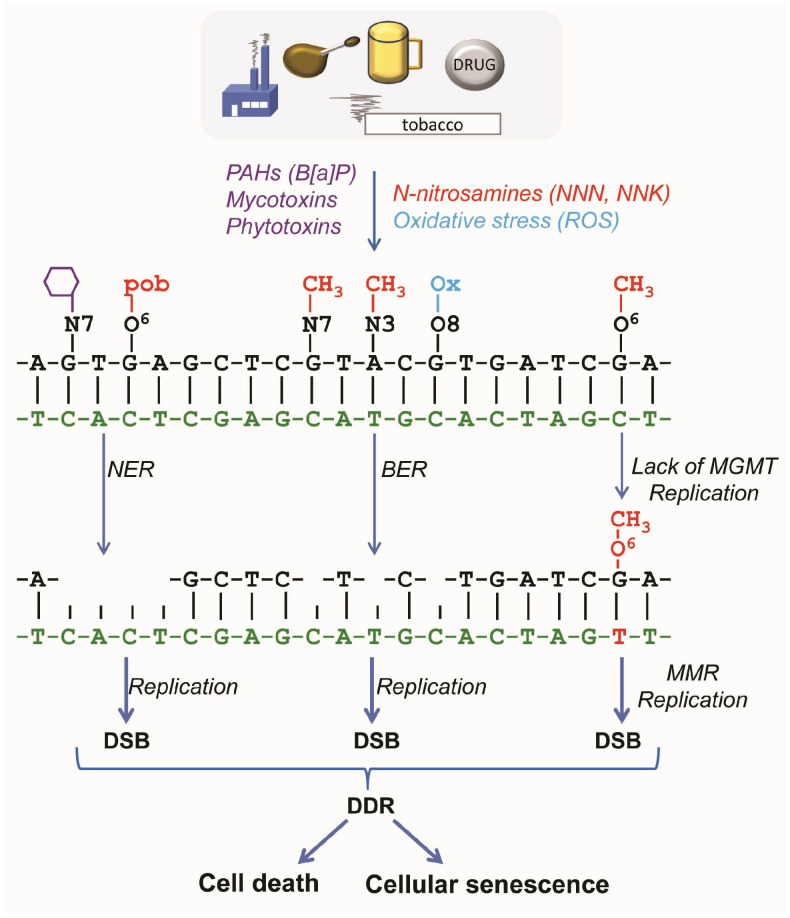
**Genotoxin-induced pathways leading to DSBs that trigger cell death and CSEN.** Carcinogens can induce multiple primary DNA adducts, which are subject to repair by nucleotide excision repair (NER), base excision repair (BER), and the suicide enzyme O^6^-methylguanin-DNA methyltransferase (MGMT). In the case of BER and NER, the repair leads to single-strand gaps in the DNA, which can lead to the formation of DSBs when encountering replication forks. In the case of MGMT, the absence of repair can lead to DSBs via mismatch repair (MMR) and replication.

There are several sources of DSB formation. They may be formed (a) directly by IR or through inhibition of enzymes involved in DNA metabolism, such as topoisomerase II, or (b) as a result of replication of a damaged template through nuclease attack at stalled replication forks or collision of the replication machinery with base excision repair (BER) or mismatch repair (MMR) intermediates [12,13]. For the specific DNA alkylation damage O^6^-methylguanine (O^6^-MeG) induced by genotoxic N-nitrosamines, the pathways have been elucidated in detail. O^6^-MeG is a very potent inducer of apoptosis and CSEN. However, it needs DNA replication and processing via mismatch repair, resulting in secondary lesions (DNA gaps and stalled replication forks) that are subsequently converted into DSBs, which are the ultimate trigger of DDR, apoptosis, and CSEN (Figure 2). Since the process requires DNA replication, only replicating cells are affected. In quiescent cells, the lesion is ineffective [14]. The same applies to bulky lesions, which lead to DSBs during replication that activate DDR [15]. Of note, DDR activation can also occur in quiescent cells, likely due to blocked transcription sites [16]. However, higher doses are very likely required for triggering cell death and CSEN in quiescent cells [17].

## 3. Molecular Mechanisms of DNA Damage-Triggered CSEN

**Figure 3:**
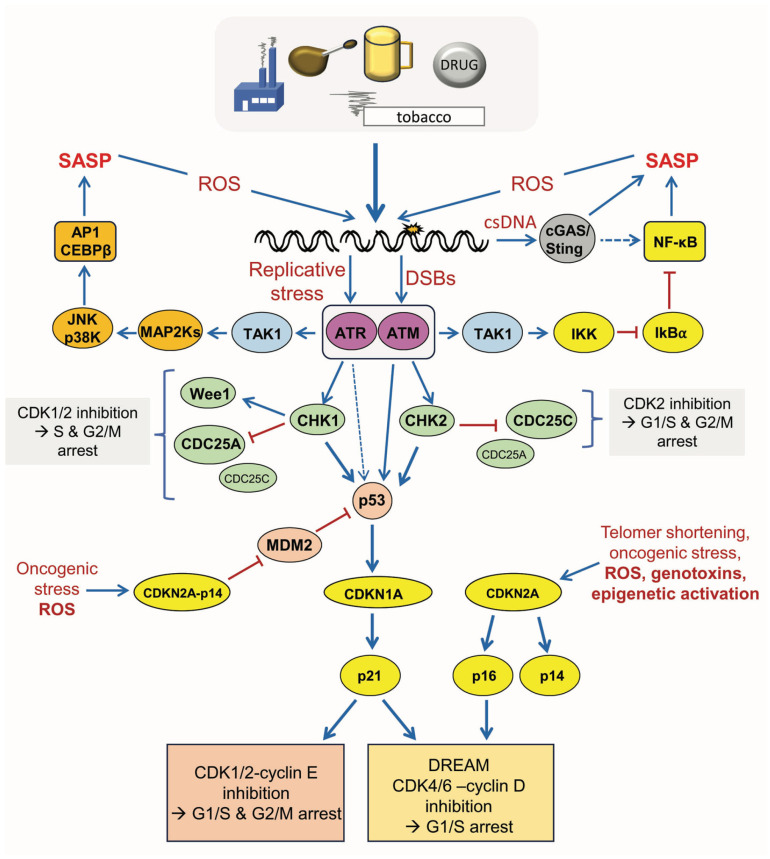
**CSEN pathways triggered by exogenous genotoxins.** The kinases ATR and ATM are activated by replication stress and DSBs, respectively, and trigger cell cycle arrest by phosphorylation of important downstream targets. ATR predominantly phosphorylates and thereby activates checkpoint kinase 1 (CHK1), which in turn activates WEE1 and, at the same time, inactivates CDC25A. This results in inactivation of the cyclin-dependent kinases CDK1 and CDK2, which causes a lack of Rb phosphorylation, inactivation of S-phase regulating E2Fs, and, finally, the arrest of cells predominantly in the S- and G2/M phase. On the other hand, ATM predominantly phosphorylates/activates checkpoint kinase 2 (CHK2), which inactivates CDC25C, thereby inactivating CDK2 and predominantly inducing G1/S and G2/M arrest. In addition, ATM and CHK2 are potent activators of p53 through phosphorylation at different sites, thus preventing its degradation by MDM2. Accumulated and activated p53 provokes transcriptional activation of the gene encoding CDK inhibitor p21 (*CDKN1A*). The p21 protein binds to and inhibits CDK2-cyclin E/A and CDK1-cyclin B complexes, enforcing arrest at both the G1/S and G2/M transitions. Moreover, p21 can bind to and inhibit CDK4–cyclin D and CDK6–cyclin D complexes, inducing a DREAM-dependent G1/S arrest. Besides p21, two additional CDK inhibitors are involved in CSEN, p14 and p16. Both factors encoded by the same gene (*CDKN2A*) are activated upon oncogenic stress, telomer shortening, and excessive, persistent DNA damage. While p14 stabilizes p53 by blocking MDM2, p16 directly binds to CDK4 and CDK6 and thus prevents them from binding to cyclin D. This inhibits CDK4-cyclin D and CDK6-cyclin D complexes, which are not able to phosphorylate the E2F-bound Rb protein anymore. This causes E2F to remain in an inactive state, and genes required for S-phase progression are not transcribed. The *CDKN2A* gene can also be activated by genotoxic stress, which is, however, a late response and occurs in an indirect way through demethylation of the silenced gene. Thus, p16 is responsible for the maintenance of the senescent state. If damage persists, the prolonged cell cycle arrest can cause further genomic reprogramming and transition into the ultimate cellular senescence program. Besides the classical DDR, ATM/ATR can activate TAK1, which in turn activates the transcription factor NF-κB and, via p38 kinase and Jun kinase (JNK), the transcription factors AP1 and CEBPβ. These transcription factors activate the SASP, which also leads to enhanced ROS production. Moreover, the SASP can also be activated in response to csDNA via the cGAS/Sting pathway.

The DNA damage response is crucial for the induction of genotoxin-induced cell cycle arrest and CSEN; the major pathways in this complex scenario are described in more detail below (see also legend of Figure 3). First is the detection of DSBs and blocked replication forks. In brief, upon induction of DSBs, the MRN complex, consisting of MRE11, RAD50, and NBS1, binds to them [18]. MRE11 exhibits exo- and endonuclease activity, RAD50 has DNA-binding capabilities, and NBS1 is responsible for shuttling the MRN complex to the nucleus [19]. ATM is then recruited to the DSB, whereupon it becomes activated through autophosphorylation [20,21]. Once activated, ATM phosphorylates more than 500 downstream targets [22], including checkpoint kinase 2 (CHK2), which plays a role in checkpoint activation [23], histone 2AX (H2AX), which plays a role in DNA repair [24], and mediator of DNA damage checkpoint 1 (MDC1), which is important in the choice between repair by NHEJ or HR [25], among others.

Apurinic/apyrimidinic sites and bulky adducts block the DNA polymerase, which leads to their uncoupling from the helicase and the formation of long stretches of single-stranded DNA (ssDNA) at the replication fork. These are extreme fragile structures that need to be protected. This occurs by replication protein A (RPA), which then recruits the kinase ATR complexed with ATR-interacting protein (ATRIP) [26,27,28]. The 9-1-1 complex, consisting of Rad9, Hus1, and Rad1 [29], and topoisomerase II binding protein 1 (TopBP1) are then recruited [30,31]. TopBP1 directly activates the ATR-ATRIP complex, most likely via a conformational change of ATR-ATRIP [32]. ATR, in turn, phosphorylates a number of proteins, including the checkpoint kinase 1 (CHK1) [33], histone 2AX (H2AX) [34], and BRCA1 [35], which contributes to DSB repair.

*The p53 response:* The transcription factor p53, activated following genotoxic exposure, has a “Janus face”, playing a protective role by increasing DNA repair and a sensitizing role by stimulating apoptosis and CSEN. Key in the regulation of CSEN is *CDKN1A*, encoding the p21^CIP1^ protein (here shortly designated as p21), which is an inhibitor of cyclin-dependent kinases (CDK). Although CSEN can also be triggered via p53-independent pathways following genotoxic stress, there is a consensus that p53 is at the top of the decision between survival, death, and CSEN. How is this decision made? Much of the answer is speculative, but it seems that most important is the way p53 is modified. p53 can be phosphorylated at different sites (Ser6, Ser9, Ser15, Ser20, Ser37, Ser46, or Ser392). p53-Ser15 and -Ser20 are considered to activate the *CDKN1A* gene, and, therefore, regulate CSEN through the upregulation of p21. With a high DNA damage level, another p53 site comes into play: Ser46 [36]. The key player in this case is the kinase HIPK2, which phosphorylates p53 at this site. HIPK2 is normally complexed with the ubiquitin ligase Siah-1. This protein is, like CHK1 and CHK2, a target of ATM and ATR, which phosphorylate it and lead to HIPK2 activation with the consequence of p53 phosphorylation at Ser46 [37]. Importantly, p53^Ser46^ does not transactivate the *CDKN1A* gene and, therefore, it does not directly contribute to CSEN. However, it regulates proapoptotic genes (*PUMA* and *NOXA)* and, therefore, it may be considered a switch maker between CSEN and apoptosis upon genotoxic stress.

*p53-CDKN1A/p21-axis:* Following phosphorylation by CHK1 or CHK2, p53 accumulates in the nucleus and binds to the promoter of *CDKN1A*, thus stimulating the transcription of the gene. The encoded p21 protein acts as an inhibitor of the CDK/cyclin complexes, which, under non-stress conditions, phosphorylate the retinoblastoma (Rb) protein, leading to liberation of E2F transcription factors that activate S-phase dependent genes (for review see [38,39]). This cannot occur under stress conditions, leading to transient and chronic proliferation arrest, which is a hallmark of CSEN. Most importantly, induction of p21 can activate the gene repressor–DREAM complex via inhibition of CDK4-Cyclin D and, therefore, can arrest cells in different cell cycle phases [40]. The DREAM complex has been shown to be a master regulator of somatic DNA repair and CSEN [41,42].

An alternative pathway is triggered by p16^INK4A^ (briefly p16) and p14^ARF^ (briefly p14), which are encoded by the same gene, *CDKN2A*, but arise from alternative promoter usage. Transcriptional regulation upon genotoxic stress differs from p21. Following activation of ATM/ATR, E2F1 becomes more stable and transcriptionally active. It directly induces transcription of p14. In turn, p14 inhibits MDM2 and thus stabilizes p53, causing a robust p21 mediated CDK-inhibitory response, cell cycle arrest, and CSEN. In this way, p14 causes a p53 stabilization loop and increases the activity of p53 [43,44,45] thus enhancing CSEN [46].

Of note, *CDKN2A/p16*, which inhibits CDK4 and CDK6, is not a target of p53 [47]. Its induction occurs more slowly than *CDKN1A*/p21 and is more complex, involving epigenetic de-repression of the gene, activation by the p38-MAPK pathway involving c-Jun and ATF2, the Ets family transcription factors, and more (for review, see [48]). Interestingly, p53 loss or genetic inactivation accelerates p16 expression, and p21-induced cell cycle arrest fosters chromatin remodeling, allowing enhanced p16 expression [41,49]. Overall, p16 is considered important for long-term maintenance of the senescent state, especially during replicative and oncogenic stress (see Figure 3). However, upon acute genomic stress, it is dispensable for the activation of CSEN [50].

It is important to note that DDR activation following DNA damage is a process triggering both pro- and anti-survival pathways, including CSEN. Thus, downstream targets of the ATR/ATM-CHK1/CHK2-p53 (AACP) axis are pro-survival factors involved in cell cycle inhibition (p21, Wee1), DNA repair (DDB2, XPC, FEN1, and POLH) [51], and autophagy [52]. Like a double-edged sword, this axis also regulates genes involved in apoptosis, such as the death receptor Fas (Fas-R), Bax, Puma, Noxa, Apaf-1, and Pidd [37] and CSEN. How the balance between survival, death, and CSEN is regulated is not well understood. Thus, in glioblastoma cells treated with the methylating drug temozolomide (inducing the same spectrum of DNA lesions as N-nitrosamine), apoptosis, autophagy, and CSEN increase as a linear function of dose, without a detectable threshold for one of the endpoints [53]. This indicates that in the same genotoxin-exposed population, cells are ready to make the decision to either die or enter the senescence state.

**Figure 4:**
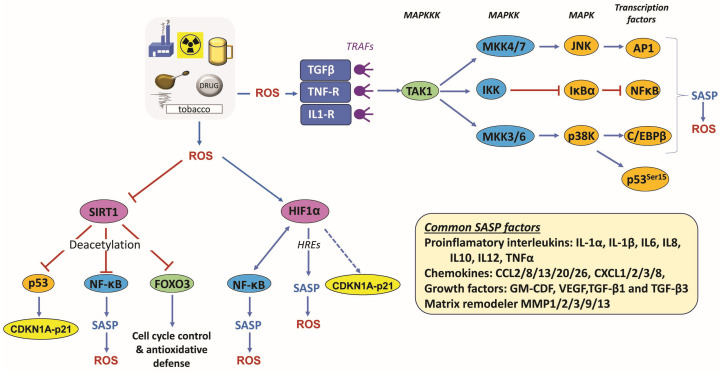
**Regulation of SASP and related responses**. Besides the activation of DDR through initial DNA damage, permanent intracellular production of oxidative stress (ROS) is involved in turning the cell cycle arrest irreversible. ROS can directly modify growth factor and interleukin receptors, leading to activation of TAK1, which in turn activates the transcription factors NF-κB and, via p38K and JNK, the transcription factors AP1 and CEBPβ. These transcription factors activate SASP. An additional target activated by ROS is HIF1α, which can indirectly activate *CDKN1A-p21* and also interplays with NF-κB. Most importantly, it directly activates multiple SASP factors via heat shock responsive (HRE) elements. Finally, ROS can inactivate SIRT1, leading to enhanced acetylation and thereby activation of p53, NF-κB and FOXO3. Activation of the SASP leads to additional production of ROS, which results in a *circulus vitiosus* maintaining CSEN and likely also inflammatory responses.

*Role of MAP kinases and NF-κB in CSEN*: Both MAP kinases, like p38K and JNK, and the transcription factor nuclear factor kappa B (NF-κB) are central regulators of CSEN, acting as signal integrators that convert stress into a stable senescence phenotype. NF-κB regulates genes involved in inflammation, immune response, cell survival, stress adaptation, and CSEN. Upon genotoxic stress, these factors are activated via ATM and the MAP kinases TAK1 or TAO (for review, see [54,55,56]). NF-κB is primarily activated by pro-inflammatory cytokines (TNF-α and ILK-1) and during DDR through NEMO [57,58], which is involved in the degradation of the inhibitor (IκBα) and release of NF-κB dimers (p65/p50) into the nucleus, inducing transcription of its target genes. In this scenario, ATM is a key driver of NF-κB-dependent DNA damage-induced CSEN, cell dysfunction, and aging [59]. Overall, NF-κB is the most important transcription factor mediating the SASP (see also Figure 4). In summary, MAPK and NF-kB are involved in CSEN regulation through a complex network and are of utmost importance for regulating SASP factors and cell cycle inhibitors.

*Role of cGAS/STING:* A specific pathway involved in the activation of CSEN is the cGAS/STING pathway [60,61]. Shortly, this pathway is activated by the binding of the cyclic GMA-AMP synthase cGAS to double-stranded DNA in the cytoplasm, which may originate from damaged DNA and disrupted micronuclei. Upon activation, cGAS uses ATP and GTP to synthesize the secondary messenger 2′3′-cyclic GMP-AMP (cGAMP). cGAMP binds to and activates STING (stimulator of interferon genes), which recruits TBK1 (TANK-binding kinase 1). In response, TBK1 phosphorylates IRF3 (interferon regulatory factor 3), which translocates into the nucleus and initiates transcription of IFN-α and IFN-β [62]. Moreover, the cGAS/STING pathway can also activate the NF-κB pathway. Thus, it was shown that STING is able to activate TAK1, and TAK1 facilitates ER exit of NF-κB [63]. Overall, the results suggest that DNA damage can activate cGAS/STING, which contributes to eliciting an immune response.

## 4. ROS Signaling and Senescence-Associated Secretory Phenotype (SASP)

Senescent cells are characterized by genetic and metabolic reprogramming. As a consequence of the activation of the broad-spectrum gene repressor complex DREAM, many genes are downregulated [40,64,65]. These include cell cycle genes and DNA repair genes, and it has been suggested that repression of DNA repair is a hallmark of CSEN [66]. On the other hand, there are also genes that are upregulated in CSEN cells, causing the senescence-associated secretory phenotype (SASP).

The SASP is an important feature of CSEN, which poses a threat to neighboring cells and thereby the whole organism. It is characterized by the secretion of multiple immune-stimulating factors, including proinflammatory interleukins (IL6, IL8), chemokines, growth factors and matrix metalloproteinases (Figure 4). The main transcription factors involved in the regulation of this process are C/EBPβ [67], NF-κB [68] c-Myc [69] and AP1 [70]. Moreover, epigenetic mechanisms are important for orchestrating SASP expression [71].

The SASP is strongly connected with an increase in ROS production and DNA damage [72]. Moreover, it enhances the proliferation of neoplastic epithelial cells [73], promotes epithelial-to-mesenchymal transition (EMT) [74] and tumor growth in vivo [75]. Of note, the SASP was also observed in non-proliferating cells as part of a senescence-like phenotype in postmitotic cells [76]. CSEN and SASP-driven chronic inflammation significantly contribute to the development or worsening of many age-associated diseases, as well as other pathologies, such as metabolic and endocrine disorders [77], atherosclerosis [78], neurodegenerative diseases [79], sarcopenia [80], cancer [81], diseases of the lung [82], kidney [83,84] and liver [85], as well as skin aging [86]. It is important to note that ROS production and SASP may be critical for the maintenance of CSEN after the initial nuclear DNA damage is repaired [87]. In conclusion, the SASP associated with chronic inflammation and DNA-damaging ROS is considered a potent tumor promoter, accelerating tumor development and progression.

Where does ROS come from? A main source is mitochondrial dysfunction, but also oncogene activation can cause cellular ROS overproduction. Another source are immunocompetent cells (monocytes, macrophages, and neutrophils) that, upon activation, respond with a ROS burst (e.g., through activation of NADPH oxidases). Therefore, inflammation triggered by CSEN is always bound to increased DNA damage in the surrounding tissue through ROS. It is worth to note that in senescent cancer cells following genotoxic therapy, a high ROS level together with a high level of oxidative DNA lesions was found (an example is given in [88]), which may trigger cancer-related SASP and tumor progression.

As a consequence of ROS overproduction, cellular receptors can be activated, such as TNF-R and TOLL-like receptors (TLRs). This leads to recruitment and binding of TNF-receptor-associated factors (TRAF2,3,6). These factors possess E3-ubiquitin ligase activity and form ubiquitin chains, which act as an anchor for TAK1-TAB1/2/3. Upon oligomerization and autophosphorylation, TAK1 finally activates the transcription factors AP1 and C/EBPβ, as well as NF-κB [89], which mediate activation and maintenance of the SASP (Figure 4).

Another important link between oxidative stress and SASP is the activation of sirtuin 1 (SIRT1) and HIF1α. SIRT1 is a NAD^+^-dependent deacetylase, which acts as a sensor and mediator of oxidative stress. It is a key factor in inflammation and CSEN [90]. Whereas mild stress activates SIRT1, severe stress inhibits it, leading to CSEN and SASP. In response to the ROS-dependent SIRT1 inactivation, targets like p53, FOXO3 and NF-κB become acetylated [91,92,93,94]. In the case of FOXO3, this leads to activation of anti-oxidant target genes like SOD2 [95] and the cell cycle regulator p21 (in complex with SMAD proteins) [96], in the case of NF-κB to the induction of SASP factors [68], and in the case of p53 to the induction of p21 [97]. Another regulator of SASP is HIF1α (see Figure 4).

Most important in this context is the finding that HIF1α can modulate the NF-κB signaling pathway and vice versa, thus HIF1α is activated by NF-κB [98]. HIF1α can induce multiple genes, including those encoding SASP factors [99] like IL1β [100] and IL6 [101], which harbor hypoxia response elements (HREs) in their promoters.

It should be noted that CSEN can represent a transient and a chronic state. Transient senescence is a beneficial process that is resolved once its task is completed. It is involved in embryonic development and wound healing and is cleared by the immune system. The SASP profile is moderate. Chronic (permanent) senescence, on the other hand, is a persistent, pathological state, which is merely a byproduct of excessive, unresolved cellular stress, notably due to DNA damage. Cells are apoptosis-resistant, and the immune system fails to remove them. The SASP profile is destructive through excessive release of inflammatory cytokines, causing a bystander effect [102,103]. Chronic CSEN is a key driver of age-related diseases. The pathways induced by exogenous genotoxins described above relate to permanent cellular senescence.

## 5. Environmental and Food-Borne Carcinogens Causing CSEN

Having described how CSEN is regulated on the molecular level, it is obvious that non-repaired DNA damage is key in triggering CSEN. Therefore, it is reasonable to hypothesize that all genotoxins are able to activate CSEN pathways. Although most studies on the induction of CSEN have been conducted on tumor cells exposed to genotoxic anticancer drugs (for a recent review, see [104]), there is an increasing amount of solid findings for environmental and dietary carcinogens. These include N-nitrosamines, polycyclic aromatic hydrocarbons (PAHs), tobacco-specific carcinogens, environmental neurotoxins, asbestos, food-borne carcinogens, heavy metals, ultraviolet light, and environmental exhaust (Table 1 and Table 2). Concerning UV-radiation, multiple markers of CSEN have been identified in the aging skin (for review, see [105,106]). For example, UV-radiation causes CSEN in mouse skin fibroblasts [107] and human keratinocytes [108]. In the latter case, CSEN was triggered by ROS overproduction, triggering p53-p21 signaling. In the following, we will focus on CSEN induced by tobacco smoke-associated carcinogens, specific environmental exposures and dietary carcinogens.

## 6. Tobacco Smoke and Other Pollutants Cause CSEN

Cigarette smoke contains a complex mixture of over 7000 chemical substances, of which 70–90 are carcinogenic or suspected of being carcinogenic. These ingredients harm humans in various ways, particularly affecting the respiratory system, the cardiovascular system, and numerous organs. The mechanisms through which they trigger cancer range from direct DNA damage to promoting chronic inflammatory processes, which significantly increase the long-term risk of cancer.

There is growing evidence that cigarette smoke gives rise to the induction of CSEN. Thus, cigarette smoke was shown to induce CSEN through triggering p53-p21 and p16 signaling in lung fibroblasts [109,110] and A549 cells [111], and via ROS-signaling and ERK1/2 signaling in lymphocytes [112]. Recently, it was reported that even cigarette side-stream smoke can induce CSEN, which was demonstrated in human skin fibroblasts [113]. Importantly, smoking accelerates the aging of the small airway epithelium [114] and consequences of smoking, like COPD and idiopathic pulmonary fibrosis, are associated with CSEN [115,116,117,118]. The most important carcinogens in tobacco smoke are polycyclic aromatic hydrocarbons (PAHs), N-nitrosamines, heavy metals, and fine dust, which have all been shown to induce CSEN (Table 1).

In this context, it is important to note that not only smoked tobacco, but also smokeless tobacco (used orally or nasally) contains tobacco-specific N-nitrosamines. These quite stable compounds are formed during the curing process of tobacco leaves from bacterial metabolism [119]. Consequently, consumption of smokeless tobacco is also associated with increased cancer incidence [120]. Although this has not yet been directly proven, it is reasonable to assume that smokeless tobacco also triggers CSEN.

### 6.1. Polycyclic Aromatic Hydrocarbons

Among the best-known carcinogenic substances in tobacco smoke are PAHs. The IARC Monographs program has reviewed experimental data for 60 different PAHs [121]. Of these, benzo[a]pyrene (B[a]P) is the only compound that was classified as a human carcinogen (group 1), whereas most other PAHs are classified as probably carcinogenic (group 2A) or possibly carcinogenic to humans (group 2B), as they are genotoxic and shown to cause skin cancer in rodents [121,122,123]. B[a]P has been found to be associated with the development of breast, esophagus, larynx, mouth, throat, kidney, bladder, pancreas, stomach, cervix, and blood cancer. B[a]P is not active on its own. It must be converted by enzymes such as CYP1A1 and CYP1B1 into reactive metabolites, particularly the highly reactive benzo[a]pyrene-7,8-diol-9,10-epoxide (BPDE). BPDE covalently binds to DNA and forms adducts, notably N7-guanine adducts. If these adducts are not properly repaired, they cause a hindrance for DNA enzymes and block replication and transcription. BPDE-adducts are considered to be the primary trigger of CSEN, which occurs through activation of the p53-p21 cascade [124,125,126]. Also, activation of the DREAM complex was reported [41]. Interestingly, induction of CSEN was accompanied by AhR-dependent signaling, which can lead to histone H4K12 lactylation in the vicinity of the p53 promoter [124]. B[a]P can also lead to inhibition of TERT transcriptional [127,128], which may lead to telomere shortening and CSEN.

### 6.2. N-Nitrosamines

*N*-nitroso compounds are ubiquitously distributed in the environment. They are present in food and beverages, in polluted air and water, and also in tobacco [119]. Moreover, they are endogenously produced in the stomach (from nitrite and secondary amines) and gut (from bacterial metabolism).

The most important N-nitrosamines in tobacco smoke (and in smokeless tobacco) are N-dimethyl-N-nitrosamine (DMNA, synon. N-nitroso-N-dimethylamine NDMA), 4-(methylnitrosamino)-1-(3-pyridyl)-1-butanone (NNK) and *N*′-nitrosonornicotine (NNN) [129,130]. Whereas NNN and NNK are classified as carcinogenic to humans (group 1), DMNA is classified as probably carcinogenic to humans (group 2A) [131,132]. Similar to PAHs, *N*-nitrosamines need metabolic activation to cause DNA damage. Thus, NDMA is metabolized by Cyp1A1, generating intracellular methyldiazonium ions, which methylate nucleophilic centers and form predominantly N7-methylguanine (N7-MeG), N3-methyladenine (N3-MeA), N3-methylguanine (N3-MeG) and *O*^6^-methylguanine (*O*^6^-MeG) [133]. The tobacco-specific carcinogen NNK produces, upon metabolic activation, either carbenium ions or pyridyloxobutylating (pob) agents, which lead to the formation of DNA methylation adducts (N7-MeG, N3-MeA, N3-MeG, *O*^6^-MeG, *O*^4^-MeG) and various pob-adducts, with O(6)-[4-oxo-4-(3-pyridyl)butyl]guanine (*O*^6^-pobG) being among them [134]. In contrast, NNN induces only pob-adducts after metabolization, with *O*^6^-pobG being among them [135,136]. Some of the mutagenic adducts are repaired by MGMT, which is a key genotoxic defense mechanism [137].

Data concerning CSEN induced by N-nitrosamines are limited, and only data for *N*-nitrosodiethylamin (NDEA) are available. NDEA has been found in tobacco smoke [138] and is classified as a group 2A carcinogen [139]. Thus, markers of CSEN were found in the liver of mice exposed to NDEA [140], and weekly intraperitoneal injection of NDEA induced CSEN in the liver (both in fibrotic septa and in hepatocytes) during hepatocarcinogenesis in rats [141]. The pob-adducts induced by NNK and NNN represent bulky lesions, similar to the adducts induced by B[a]P and the mycotoxins aflatoxin B1 and ochratoxin A, which were shown to induce CSEN. Since the adducts block replication, it is very likely that they activate the DDR-dependent CSEN program, which however has to be proven experimentally.

An important pre-mutagenic adduct induced by DMMA and NNN is O^6^-MeG. This lesion is repaired by MGMT, and comparison of repair competent versus repair deficient cells and mice revealed it to be the major trigger of gene mutations, cancer initiation, and cancer progression, as well as cytotoxicity (for review, see [142]). The cytotoxic response was of particular interest in the context of cancer therapy by methylating agents and, therefore, intensively studied. It was shown that O^6^-MeG is a powerful apoptosis-inducing lesion, with mismatch-repair mediated DSBs critically involved. Although the apoptotic pathways have been elucidated in detail (for review, see [13]), it was puzzling why the yield of apoptosis did not exceed a particular threshold. It turned out, however, that the same O^6^-MeG-driven upstream pathway is involved in triggering CSEN, which is even the major trait in glioblastoma cells [88,143]. In this cell system the O^6^-MeG adduct level, DSBs and p53 activation (p53Ser15 and Ser46) increased linearly together with the CSEN level ([144]).

Although the findings were obtained with glioblastoma cells, there is no reason to assume that they cannot be transferred to non-tumor cells. Given these facts, O^6^-MeG-inducing N-nitrosamines can be expected to be a powerful inducer of CSEN, especially in proliferating tissues with low MGMT expression.

Given the importance of N-nitrosamines as tobacco, food, and environmental carcinogens, it is surprising that little research has been done so far on whether this group of genotoxicants induces CSEN. A reason could be that for these studies, the compounds must be metabolically activated (for which usually S9 mix is used), and cells have to be in culture for long post-incubation periods, which is limited by the toxicity of the S9 mix itself. Therefore, instead of N-nitrosamines, N-nitrosamides such as N-methyl-N-nitrosourea (MNU) and N-methyl-N′-nitro-N-nitrosoguanidine (MNNG) or alkylsulfonates like methylmethane sulfonate (MMS) are frequently used in research as they do not need metabolic activation. MNNG was shown to induce CSEN in HCT-116 colon cancer cells [145] and MMS in RPE-1 cells [146]. MNNG produces a relatively high level of O^6^MeG (7% of total alkylation) in the DNA compared to MMS (0.3%), which produces mainly N-alkylations such as N7-MeG and 3-MeA. Therefore, N-alkylations may also contribute to CSEN induction. In summary, alkylating agents and bulky agents are powerful inducers of CSEN; however, experiments with exposure-relevant N-nitrosamines and normal human cells are still lacking and urgently needed.

### 6.3. Heavy Metals

Carcinogenic metals can enter the human body through industrial emissions, contaminated drinking water, food, and tobacco smoke. The most important metals classified as carcinogenic to humans (Group 1) by the IARC include arsenic, cadmium, chromium (VI) and nickel [147]. In addition, certain forms of cobalt are classified as carcinogenic (Groups 2A and 2B). Arsenic provokes cancer development through oxidative DNA damage, inhibition of DNA repair mechanisms, and epigenetic changes, and is linked to skin, lung, and bladder cancer [147]. Cadmium interferes with essential metals, generates oxidative stress, and can disrupt apoptosis mechanisms. It is linked to lung and prostate cancer [147]. Chromium (VI) causes DNA adducts, single- and double-strand breaks, and chromosomal instability, and is associated with lung cancer [148]. Lastly, nickel acts as an indirect carcinogen by modifying epigenetic patterns and disrupting gene expression [149] and is associated with nasal and lung cancer.

There is an increasing amount of data showing that heavy metals induce CSEN [150,151]. Thus, cadmium was reported to induce CSEN in rat bone marrow-derived mesenchymal stromal cells via NF-κB [152], in renal tubular HK-2 cells and rat osteoblasts via the Sirtuin-1 pathway [153,154] and in rat annulus fibrosis cells via the JNK pathway [155]. Importantly, in all studies, the p53-p21 pathway was involved. Cadmium-dependent induction of CSEN was also associated with activation of the cGAS-STING pathway in alveolar epithelial cells, with subsequent induction of chronic obstructive pulmonary disease (COPD) [156]. Also in this experimental system, the p53-p21 pathway was involved.

Arsenic was shown to activate CSEN via p53-p21 in the hepatic stellate cell line LX-2 [157] and the hepatocyte-derived cell line Huh-7 [158]. Also, for nickel, induction of CSEN was reported, which induced CSEN in multiple human lung cell lines via HIF1α-dependent upregulation of p21 and p27 [159]. Hexavalent chromium has shown to induce CSEN in L-02 hepatocytes via the ROS and p53-p21 pathway [160,161] and via the GATA4/NF-κB pathway [162]. Finally, cobalt, which acts via ROS generation and interference with DNA repair mechanisms, induced CSEN in synovial fibroblasts [163]. Taken together, heavy metals appear to be powerful inducers of CSEN with major activation of the p53-p21 pathway.

### 6.4. Fine Dust

When tobacco or wood and fossil energy are burned, particles are produced, many of which belong to fine dust of the PM10, PM2.5, and even ultrafine particle (PM0.1) classes. Especially, PM2.5 particles can penetrate deep into the lungs and induce lung cancer. Therefore, PM2.5 particles are classified as IARC Group 1 [147]. Exposure to PM2.5 particles of A549 lung epithelial cells induces CSEN via the cGAS-STING and NF-κB pathway [164], in HaCaT and HEK001 cells with the involvement of ROS signaling [165], and in HUVECs via the NF-κB/NLRP3 pathway [166]. Furthermore, ultrafine particles were shown to induce CSEN also in mouse macrophages, which was accompanied by activation of the phagolysosome–15-lipoxygenase pathway [167].

**Table 1:** **Carcinogens/genotoxins triggering CSEN. Revealed pathways and cellular systems used in the study.**

Genotoxin	Pathway	Cellular System	Reference
**Cigarette smoke**			
Cigarette smoke	p53-p21, p16	Lung fibroblasts, HFL-1	[109,110]
	p53-p21	A549 cells	[111]
	ROS-signaling, ERK1/2	Th17 lymphocytes	[112]
Cigarette sidestream smoke	-	Human skin fibroblast ASF-4-1	[113]
**Fine dust**			
Fine particles PM2.5	cGAS-STING, NF-κB	A549 lung epithelial cells	[164]
	ROS-signaling	HaCaT and HEK001	[165]
	NF-κB/NLRP3	HUVECs	[166]
Ultrafine particles	Phagolysosome–15-lipoxygenase	Mouse macrophages ex vivo	[167]
**PAHs**			
B[a]P/BPDE	Inhibition of TERT transcriptional expression	Mouse spermatocyte-derived GC-2 cells	[127,128]
	p53-p21-DREAM	MCF7 cells	[41,126]
	p53-p21	MCF7 cells	[125]
	AhR mediated H4K12 lactylation at the p53 promoter	Mouse lung epithelial MLE-12 cells	[124]
**Metals**			
Nickel	HIF-1α, p21, p27	Human H460, WI38, A549, IMR90 lung cells	[159]
Cobalt	-	Synovial fibroblasts	[163]
Arsenic	-	Hepatic stellate cell line, LX-2	[157]
Arsenite	p53-p21	Hepatocyte-derived cell line Huh-7	[158]
Chromium	ROS-signalling, p53-p21	L-02 hepatocytes	[160,161]
	GATA4/NFκB	L-02 hepatocytes	[162]
Cadmium	NF-κB, p53-p21	Rat bone marrow-derived mesenchymal stromal cells (BMMSCs)	[152]
	SIRT1, p53-p21	Renal tubular cells HK-2	[153]
	JNK, p53-p21	Rat annulus fibrosus (AF) cells	[155]
	cGAS-STING, p53-p21	Mouse lung epithelial cells	[156]
	SIRT1	Rat osteoblasts	[154]
**Additional substances**			
MNNG	-	HCT116 cells	[145]
MMS	-	RPE-1 cells	[146]
NDEA	p53-p21	Rat Hepatocytes	[141]
PVC	ROS-signalling	A549 and BEAS-2B	[168]
Ultraviolet light	-	Mouse skin fibroblasts	[107]
	ROS-signalling, p53-p21	Human keratinocytes	[108]

## 7. Dietary Genotoxins/Carcinogens and CSEN

Carcinogenic substances in food can arise through contamination with carcinogenic phytotoxins or infection with microorganisms like mold and bacteria producing mycotoxins or phytotoxins. Among food-borne toxins with established or suspected carcinogenicity are also compounds formed during cooking or food preservation. These compounds belong to the group of N-nitrosamines and PAHs (see smoking-related carcinogens). They also include products of food heating, like heterocyclic aromatic amines (HCA) and acrylamide. Finally, food can also be contaminated by environmental chemicals like pesticides and heavy metals. In addition, food and beverages can bear a carcinogenic potential if they are overconsumed. Examples are red meat and ethanol (resp. the degradation product acetaldehyde), which are classified as group 1 and group 2A carcinogens, respectively. Since PAHs, N-nitrosamines, and heavy metals have already been discussed, we will focus on the following: HCAs, acrylamide, and the harmful food contaminants mycotoxins, phytotoxins, and phycotoxins (for a compilation, see Table 2).

### 7.1. Heterocyclic Aromatic Amines (HCAs)

HCAs are formed primarily during the cooking of meat at high temperatures. Among the HCAs, IQ (2-Amino-3-methylimidazo[4,5-f]quinoline) is classified into IARC group 1, MeIQ (2-Amino-3,4-dimethylimidazo[4,5-f]quinoline), MeIQx (2-Amino-3,8-dimethylimidazo[4,5-f]quinoxaline) in group 2A and PhIP (2-Amino-1-methyl-6-phenylimidazo[4,5-b]pyridine), Trp-P-1(3-Amino-1,4-dimethyl-5H-pyrido[4,3-b]indol), Trp-P-2 (3-Amino-1-methyl-5H-pyrido[4,3-b]indole), AαC (Aα-carboline, 2-Amino-9H-pyrido[2,3-b]indole) and MeAαC (2-Amino-3-methyl-9H-pyrido[2,3-b]indole) in group 2B [169,170]. Although these compounds are potent genotoxins, for none of them a clear induction of CSEN has been shown yet. Interestingly, aerosol particles of PhIP-incorporated oleic acid, which simulate fumes from meat cooking, but not dissolved PhIP, showed SA-β-galactosidase positivity in SHSY5Y, MRC5, and human dermal fibroblast cells [171]. This may indicate that the way of exposure plays a critical role.

### 7.2. Acrylamide

Acrylamide is formed during the high-temperature cooking of starchy foods as a result of the Maillard reaction and is classified into IARC 2A, as it induces cancer in rodents [172]. It has been shown that acrylamide induces CSEN in mouse macrophages through ROS and activation of the p38/JNK pathway [173]. Also, in human endothelial cells, acrylamide and its metabolite glycidamide induced CSEN; however, the mechanism was not analyzed [174]. In embryonic fibroblasts, acrylamide-induced CSEN was associated with activation of the p38K and p53/p21 pathways [175].

### 7.3. Red Meat

Although N-nitrosamines and heterocyclic amines are also formed during the preparation of fish and white, blood-poor meat (chicken and turkey), a correlation between meat consumption and colorectal cancer incidence has only been demonstrated for red meat [176,177]. Red meat (pork, beef, lamb, and veal) is characterized by a high content of hemoglobin present in erythrocytes. At its center, hemoglobin contains an iron group, heme. This heme has been identified as a cause of colorectal cancer. Dietary heme is also present in high amounts in the myoglobin of red meat muscle cells.

Heme (and the non-protein-bound hemin) itself does not bind to DNA and is not genotoxic. The leading mechanistic hypothesis (for review, see [178]) rests on heme iron-driven formation of carcinogenic species in the gut lumen, not on intrinsic mutagenicity of hemoglobin and myoglobin. Heme can provoke genotoxin formation in three ways: First, heme iron can catalyze the Fenton reaction (H_2_O_2_ + Fe^2+^), leading to the formation of hydroxyl radicals (OH^•^) that damage the DNA, causing the formation of the mutagenic 8-oxo-guanine, abasic sites, and DNA breaks [179]. Second, heme strongly promotes lipid peroxidation in gut lumen cells, producing malondialdehyde and 4-hydroxynonenal (4-HNE), which are clearly genotoxic and mutagenic [180]. Third, heme enhances the endogenous formation of N-nitrosamines and N-nitrosamides [181], which cause the formation of N- and O-alkylation adducts and DSBs following replication. In summary, overconsumption of red meat can cause a pro-oxidant and pro-nitrosating microenvironment in the colon, driven by heme iron. This explains the tissue-specific (colon) risk enhancement. It further explains the findings that fiber, calcium, and antioxidants mitigate the risk, and that white meat (low heme) does not show the same cancer-provoking effect.

In light of these results, it is appropriate to ask whether red meat (heme) can also induce CSEN. This seems to be very likely as heme induces DNA damage, which meets the paradigm that DNA damage (especially DSB) activates the DDR, which triggers not only apoptosis but also CSEN. If the DNA damage is persistent and sublethal, cells may preferentially enter premature senescence rather than apoptosis. Actually, 4-HNE is a well-established CSEN inducer activating the p53-p21 axis and promoting p16 [182]. Further, in vitro heme/hemin was shown to induce CSEN markers, including SA-ß-gal positivity, p21 and p16 induction, G1 arrest, and flattened cell morphology [183]. Importantly, at low/moderate doses, CSEN was induced; at high doses, apoptosis or ferroptosis occurred. In vivo, hemin was shown to induce CSEN via DDR in neurons [183]. It will be of interest to see whether colon epithelial cells show accumulation of senescent cells in individuals upon chronic high red meat consumption.

In summary, heme is a strong candidate driver for CSEN in the gut. In this context, it should be mentioned that heme induces gut epithelial injury and inflammation. Senescent colonocytes may promote inflammation through the secretion of SASP factors (IL-6, IL-8, PGE_2_), which may promote carcinogenesis. Verification was provided in a study showing that chronic intestinal inflammation drives colorectal tumor formation, which is fostered by dietary heme iron in mice [184]. Therefore, heme-induced CSEN is biologically relevant as it may contribute to chronic gut inflammation, gut dysfunction, and tumor promotion.

### 7.4. Mycotoxins

Mycotoxins (mold-derived toxins) are mainly found as contaminants in grains, nuts, spices, coffee, and milk. Among those, aflatoxins (B_1_, B_2_, G_1_, G_2_, and M_1_) produced by molds of the genus *Aspergillus* have been shown to induce liver cancer (belonging to IARC Group 1). Fumonisin B_1_, which is produced by molds of the genus *Fusarium,* has been shown to induce esophageal and liver cancer (IARC Group 2B). Ochratoxin A, produced by molds of the genera *Aspergillus* and *Penicillium,* has been shown to induce kidney cancer (IARC Group 2B), and Sterigmatocystin, produced by molds of the genus *Aspergillus,* has been shown to induce liver cancer (IARC Group 2B) [170,185].

Besides its importance, the data linking aflatoxins to CSEN are limited, and in most cases, activation of the DDR or induction of inflammation is shown as the sole marker for CSEN. Thus, aflatoxin B1 (AFB_1_) was reported to induce an S-phase arrest accompanied by an increase in p14, p16, and p27 in mouse neuronal cells [186]. AFB_1_ was also able to enhance the production of inflammatory factors through NF-κB in alveolar epithelial cells [187]. Moreover, dietary AFB_1_ provoked overexpression of p21 and, in turn, arrested cells in the cell cycle via inhibiting the CDK4 interaction with cyclin D1 in mice testicular tissue [188] or by cyclin D1/CDK6 repression in chicken splenocytes [189]. Although in these studies CSEN was not directly measured, it is likely that the responses were translated into permanent cell cycle arrest. This was actually shown in a subsequent study, revealing that AFB_1_ exposure induced CSEN in mouse skin fibroblasts in vitro and accelerated skin aging in vivo, as indicated by an elevated SA-β-gal activity, p53, p21, and p16 levels. Moreover, the importance of ROS in this process was confirmed [190].

Ochratoxin A was reported to induce CSEN (measured by the β-galactosidase assay) and SASP in human renal proximal tubular cells, which was associated with p53/p21 and p16 signaling [191]. Similar results were also obtained in ochratoxin A-exposed rat intestinal crypt epithelial cells [192]. Moreover, transcriptomics revealed cell cycle regulation and CSEN as key outcomes of ochratoxin exposure [193,194].

Concerning deoxynivalenol (DON), early studies identified hints of CSEN, like increased expression of SASP factors [195,196] and induction of a p21-dependent cell cycle arrest [197]. Recently, it was shown to induce CSEN in RAW264.7 macrophages via HIF1α-mediated activation of the p53/p21 pathway [198].

Alternariol (AOH) provoked changes in cell morphology, increased β-galactosidase activity, cell cycle arrest, and expression of pro-inflammatory factors, which were observed in RAW264.7 and primary macrophages, indicating its potential as an inducer of CSEN [199,200].

Fumenosin B1 (FB1) exposure of chicken hepatocytes led to cell proliferation inhibition, cell cycle disorder, and accelerated CSEN as measured by β-galactosidase positivity [201]. In this study, CSEN was dependent on mitophagy.

The T-2 toxin represents another mycotoxin produced by *Fusarium* species (classified into IARC group 3) [170]. Initially, it was shown that it activates the p53/p21 and p16 pathway, leading to downregulation of cyclinD1 and CDK4 and subsequent cell cycle inhibition in rat pituitary tumor-derived GH3 cells [202]. Recently, it was further shown that the T-2 toxin also activates the p53/p21 and p16 pathway, causes cell cycle arrest induces SASP factors (IL-8, IL-6, and CCL2) in human neuroblastoma SH-SY5Y cells and RAW264.7 macrophages [203,204]. In these studies, the importance of the HIF1α/cGAS-STING axis for these phenotypes was demonstrated. SA-β-galactosidase positivity confirmed that these effects were caused by the induction of CSEN.

Zearalenone represents another product of the *Fusarium* species, which has not yet been classified by the IARC. Nevertheless, it clearly induces CSEN of cardiovascular cells in vitro and in vivo [205]. This is highly interesting since zearalenone is not considered to be directly carcinogenic but bears pseudo-estrogenic activity. However, the compound is also able to activate p16 and p21, which is in line with data reporting genotoxicity of zearalenone [206].

### 7.5. Phytotoxins

Phytotoxins (plant-derived toxins) are present in foods, herbal products and traditional diets. Multiple carcinogenic compounds have been found [207,208,209]. Among them are aristolochic acid, which induces kidney and urinary tract cancer, and arecoline from areca nut (betel nut), which causes oral cancer (IARC group 1). Other phytotoxins are pyrrolizidine alkaloids and safrole, which induce liver cancer (group 2B), and ptaquiloside (from bracken fern), which induces gastric and bladder cancer (group 2B).

It is established knowledge that aristolochic acid induces DNA damage in tubular epithelial cells of the kidney and causes Balkan nephropathy and Chinese herb nephropathy. These diseases are associated with enhanced CSEN in the damaged organ. Recently, it has been directly shown that aristolochic acid induces CSEN in the mouse kidney by means of SA-β-galactosidase positivity and p16 activation [210].

Among the pyrrolizidines, retrorsine is able to induce CSEN (measured by SA-β-galactosidase and p21/p27 activation) [211], and following exposure of cells to lasiocarpine, a G2/M arrest and altered morphology indicating CSEN were observed [212].

Moreover, arecoline and arecaidine were reported to induce CSEN in oral fibroblasts (SA-β-galactosidase and p16 induction) [213] and in the epithelium of oral submucous fibrosis, arecoline induced SA-β-galactosidase and the p53/p21 pathway, indicating CSEN [214]. For safrole and ptaquiloside, data concerning CSEN are not available.

### 7.6. Phycotoxins

Phycotoxins (cyanobacterial toxins) are mainly found as contaminants in drinking water, fish and shellfish. Among them is microcystin, which has been shown to induce liver cancer (IARC group 2B) [215]. Among other prominent phycotoxins, nodularin has not yet been classified; however, it is considered potentially carcinogenic due to a strong structural and mechanistic similarity to microcystin and its tumor-promoting activity in animal models. Moreover, cylindrospermopsin is genotoxic, whereas saxitoxin, brevetoxins, ciguatoxins, and anatoxin-a are highly toxic but not considered carcinogenic. For phycotoxins, direct experimental evidence for classic CSEN markers (such as increased SA-β-gal activity) is not yet available. However, several studies show DNA damage, cell cycle arrest pathways, and stress responses. For example, microcystin-LR was shown to induce the p53/p21 pathway in human peripheral blood lymphocytes [216], and cylindrospermopsin induced p53/p21 and a G0/G1 arrest in HepG2 cells [217,218].

**Table 2:** **Genotoxic/carcinogenic food contaminants inducing CSEN.**

Genotoxin	Pathway	Cellular System	Reference
**Mycotoxins**			
Deoxynivalenol	HIF1α, p53-p21, p16	RAW264.7 macrophage	[198]
Ochratoxin A	p53-p21, p16	Human renal proximal tubular cells	[191]
	ROS-signalling, p53-p21, p16	Rat intestinal crypt epithelial cells	[192]
Aflatoxin B1	ROS-signalling	mouse skin fibroblasts L929 cells	[190]
Zearalenone	p16, p21	Cardiomyocyte cell lines and primary Coronary endothelial cells	[205]
Fumenosin B1	Mitophagy	Chicken hepatocytes	[201]
Alternariol	-	RAW 264.7 primary macrophages	[199,200]
T-2 toxin	p53-p21, p16 HIF-1α/cGAS-STING	Neuroblastoma SH-SY5Y cells and RAW 264.7 macrophages	[203,204]
**Phytotoxins**			
Retrorsine	p21/p27	Rat hepatocytes	[211]
Aristolochia acid	p16	Mouse kidney	[210]
Lasiocarpine	WEE1, CHEK1 repression	V79(3A4), HepG2(3A4)	[212]
Arecoline	p16	Oral fibroblast	[213]
	p53/p21	Epithelium of oral fibrosis	[214]
**Additional substances**			
Acrylamide Glycidamide	-	HUVECs	[174]
	ROS, p38K/JNK	Mouse macrophages	[173]
	p38K, p53/p21	NIH-3T3	[175]

In summary, for most dietary carcinogens derived from food contamination, a potential for CSEN induction has been shown, at least using in vitro cell systems. Thus, nearly all carcinogenic mycotoxins examined so far were shown to induce CSEN, mostly via the p53/p21 and p16/p14 pathways. For phytotoxins, evidence was provided for aristolochic acid (via p16), the pyrrolizidine alkaloid retrorsine (via p21/p27), and the betel nut-derived arecoline and arecaidine (via p16 and p53/p21). For phycotoxins, activation of the p53/p21 pathway and cell cycle arrest have been shown for microcystin and cylindrospermopsin. Also, acrylamide and B[a]P have been shown (in vitro) to induce CSEN via p38K and p53/p21. For acrylamide, it should be noted that it is believed to exert its effect through metabolic activation into glycidamide, which generates mostly base N-alkylations. At low (exposure) concentrations, there is conflicting evidence for direct genotoxicity/mutagenicity [219]. Regarding carcinogens formed during cooking or food preservation, the results are not convincing. Similar to N-nitrosamines, no reliable data is available for HCAs (presumably due to technical issues). Therefore, research on their CSEN activating potential is highly warranted.

Finally, some open questions should be addressed. First, it is important to point out that almost all studies to date to identify the CSEN-inducing potential of food and environmental genotoxins have been conducted using in vitro cultured cells. Based on these data, the primary goal of this review was to demonstrate that CSEN can be activated by specific treatments via specific cellular pathways, and to emphasize the associated molecular mechanisms. However, the transferability of these findings to the in vivo situation is another matter. This is where the question of dose dependence comes into play. Are genotoxin exposures to which humans are subjected high enough to induce CSEN, comparable to in vitro experiments? This question can only be addressed through in vivo dose-response experiments and epidemiological studies using biomarkers in humans. It is important to note that in experimental studies, single doses are applied, while in the real life individuals are usually chronically exposed (for example tobacco, alcohol, and air pollutants). An increasing number of studies associate consequences of smoking, like COPD and idiopathic pulmonary fibrosis, with CSEN [115,116,117,118].

Tightly related to this is the question of possible threshold doses for CSEN induction. Two scenarios may be discussed. Firstly, it is conceivable that in cells with unrepaired DNA damage, low doses induce transient cell cycle arrest to enable DNA repair, higher doses induce senescence, and further dose increase leads to cell death, for example, through apoptosis. This is a scenario we logically imagine and for which evidence can be provided for specific carcinogens (our unpublished data for B[a]P). On the other hand, using glioblastoma cells treated with the alkylating drug temozolomide, we surprisingly found that CSEN and apoptosis increased linearly with the dose, without a significant threshold [220]. If we apply this data to the in vivo situation, it means that even low doses of genotoxins can induce CSEN. However, data obtained with cancer cell systems in which regulatory pathways are frequently changed and even disrupted cannot necessarily be transferred to normal cells.

Another question is whether CSEN can also be induced in non-proliferating cells by DNA damage. Since CSEN relies on the permanent activation of DDR, which is activated by DSBs and blocked replication forks, its induction by chemical genotoxins that do not directly generate DSBs is expected to be ineffective in quiescent cells. Obviously, more studies on different cell types and genotoxins are needed to elucidate the potential of acute and chronic genotoxic exposures in different cell systems to induce CSEN in humans.

An important aspect of genotoxin-induced senescence is the fact that critical DNA damage can be pre-replicatively removed by DNA repair. Damage removal is sometimes so effective that no lesions are left behind, resulting in a threshold dose, which has been demonstrated experimentally using MGMT transgenic and knockout model systems [221,222]. It is important to note that in the case of the repair enzyme MGMT, repair-competent cells are strongly resistant to CSEN (and apoptosis) induction following alkylation. MGMT is variably expressed in different organs [223], and thus it can be concluded that in organs with high expression, such as the liver, a protective effect against CSEN can be achieved through DNA repair. In organs with very low MGMT expression, such as the brain, this protective effect is very likely attenuated. Further experimental studies are needed to consolidate these inferences.

## 8. Conclusions

The list of exogenous DNA-damaging agents is long. It includes chemical and physical toxins to which we are all permanently exposed. The exposure and the degree of DNA damage depend mostly on dietary habits and lifestyle, and are influenced by epigenetic and genetic factors determining the DNA repair capacity and defense functions. Although exogenous genotoxins induce a variety of different primary DNA lesions, they lead directly or indirectly during DNA replication to DNA breaks (most important are DSBs). These, as well as replication and repair intermediates in arrested replication forks, cause activation of DDR, which includes the CDK inhibitory proteins p21 and p16 (encoded by *CDKN1A* and *CDKN2A*, respectively) as key elements. Senescent cells are characterized by a high degree of DNA damage, which permanently activates the DDR and metabolic reprogramming. In this light, CSEN is a metabolic state driven by unrepaired DNA damage, and DNA repair counteracts CSEN. DNA repair is therefore an important protective factor against CSEN and aging, which is consistent with current knowledge obtained from different experimental systems, DNA repair-deficient disorders, and epidemiology [224]. Since the number of senescent cells increases with age in the human body and is considered a driving force of aging, it can be concluded that genotoxins in the environment and in food not only influence mutation frequency and carcinogenesis but also promote the aging process itself. In light of this, measures to eliminate senescent cells or suppress the pro-inflammatory SASP are desirable. In recent years, a number of natural senolytics and senomorphics have been identified that, when taken as supplements, may eliminate or functionally limit senescent cells [225], which might be harnessed in cancer therapy [144]. Given the importance of genotoxin-triggered processes culminating in CSEN and aging, and the emerging role of therapy-induced CSEN in cancer progression and therapy as well as the role of senolytics [226], intensive research in this growing and fascinating area is warranted.

## Data Availability

No new (unpublished) data were created or analyzed in this study. Data sharing is not applicable to this article.
